# Microsecond melting and revitrification of cryo samples: protein structure and beam-induced motion

**DOI:** 10.1107/S205979832200554X

**Published:** 2022-06-14

**Authors:** Oliver F. Harder, Jonathan M. Voss, Pavel K. Olshin, Marcel Drabbels, Ulrich J. Lorenz

**Affiliations:** a Laboratory of Molecular Nanodynamics, Ecole Polytechnique Fédérale de Lausanne (EPFL), CH-1015 Lausanne, Switzerland

**Keywords:** microsecond time-resolved cryo-EM, laser melting, revitrification, single-particle reconstructions, beam-induced motion

## Abstract

A novel approach to time-resolved cryo-electron microscopy (cryo-EM) has recently been introduced that involves melting a cryo sample with a laser beam to allow protein dynamics to briefly occur in the liquid, before trapping the particles in their transient configurations by rapidly revitrifying the sample. With a time resolution of just a few microseconds, this approach is notably fast enough to study the domain motions that are typically associated with the activity of proteins but which have previously remained inaccessible. Here, crucial details are added to the characterization of the method. It is shown that single-particle reconstructions of apoferritin and Cowpea chlorotic mottle virus from revitrified samples are indistinguishable from those from conventional samples, demonstrating that melting and revitrification leaves the particles intact and that they do not undergo structural changes within the spatial resolution afforded by the instrument. How rapid revitrification affects the properties of the ice is also characterized, showing that revitrified samples exhibit comparable amounts of beam-induced motion. The results pave the way for microsecond time-resolved studies of the conformational dynamics of proteins and open up new avenues to study the vitrification process and to address beam-induced specimen movement.

## Abstract 2

Microsecond melting and revitrification of cryo samples preserves the structure of embedded particles. The beam-induced motion of revitrified samples is comparable to that of conventional cryo samples.

## 1. Introduction

Cryo-electron microscopy (cryo-EM) has been undergoing a stunning development in recent years, fueled by the introduction of a number of crucial innovations. Improved sample-preparation methods (Russo & Passmore, 2014 ▸; Naydenova *et al.*, 2020 ▸; Dandey *et al.*, 2020 ▸; Ravelli *et al.*, 2020 ▸), ever more powerful electron microscopes (Nakane *et al.*, 2020 ▸; Yip *et al.*, 2020 ▸), faster and more sensitive electron detectors (Li *et al.*, 2013 ▸) and more sophisticated computational tools (Punjani *et al.*, 2017 ▸; Zivanov *et al.*, 2020 ▸) have recently made it possible to obtain reconstructions of proteins at atomic resolution (Nakane *et al.*, 2020 ▸; Yip *et al.*, 2020 ▸). At its current rate of growth, cryo-EM is predicted to rival X-ray crystallography as the most popular method in structural biology in just a few years (Hand, 2020 ▸; Callaway, 2020 ▸). Another exciting frontier in cryo-EM is the study of dynamics, which can reveal insights into the function of a protein beyond the information that is available from a static structure (Frank, 2004 ▸, 2017 ▸; Chen & Frank, 2016 ▸). Conformational sorting with advanced computation tools can be used to map out the free-energy surface that a protein explores under equilibrium conditions in great detail (Schwander *et al.*, 2014 ▸; Dashti *et al.*, 2014 ▸; Zhong *et al.*, 2021 ▸). However, short-lived intermediates or transient states as well as fast out-of-equilibrium processes are more difficult to access. Time-resolved cryo-EM enables the study of such processes in principle, where dynamics are typically initiated with a light pulse (Ménétret *et al.*, 1991 ▸; Shaikh *et al.*, 2009 ▸) or through rapid mixing (Lu *et al.*, 2009 ▸; Shaikh *et al.*, 2014 ▸; Berriman & Unwin, 1994 ▸). The sample is plunge-frozen as the dynamics occur, trapping intermediates that can subsequently be imaged (Chen & Frank, 2016 ▸; Frank, 2017 ▸; Berriman & Unwin, 1994 ▸; Unwin & Fujiyoshi, 2012 ▸). However, the time resolution of this approach is several milliseconds and is fundamentally limited by the timescale of plunge-freezing, which is about 1 ms (Frank, 2017 ▸; Shaikh *et al.*, 2009 ▸). It is therefore too slow to capture many relevant processes, in particular the domain motions of proteins that are typically associated with their activity and that frequently occur on the microsecond to millisecond timescale (Henzler-Wildman & Kern, 2007 ▸; Boehr *et al.*, 2006 ▸).

We have recently established a novel approach to time-resolved cryo-EM that affords a microsecond time resolution (Voss *et al.*, 2021*a* ▸,*b* ▸). We illuminate a cryo sample with a focused laser beam to locally melt it and thus allow particle dynamics to briefly occur in the liquid. After tens of microseconds, the heating laser is turned off and the sample rapidly cools and revitrifies, arresting the particles in their transient configurations, in which they can be subsequently imaged. Importantly, the time resolution of our approach is determined by the timescale of vitrification, which occurs within just a few microseconds (Voss *et al.*, 2021*b* ▸). Our method thus affords a time resolution that is three orders of magnitude higher than in conventional time-resolved experiments (Shaikh *et al.*, 2009 ▸; Frank, 2017 ▸), which makes it possible to study a wide range of fast dynamics that have previously remained inaccessible.

Here, we add crucial details to the characterization of our method. While we have previously shown that the melting and revitrification process leaves the proteins intact (Voss *et al.*, 2021*a* ▸,*b* ▸), here we corroborate this finding by demonstrating that the reconstructions obtained from conventional and revitrified cryo samples are indistinguishable within the spatial resolution of our instrument. Moreover, we analyze how the revitrification process alters the properties of the ice, showing that revitrified samples exhibit similar amounts of beam-induced motion as conventional samples, albeit with small differences in the drift behavior.

## 2. Methods

**Figure 1:**
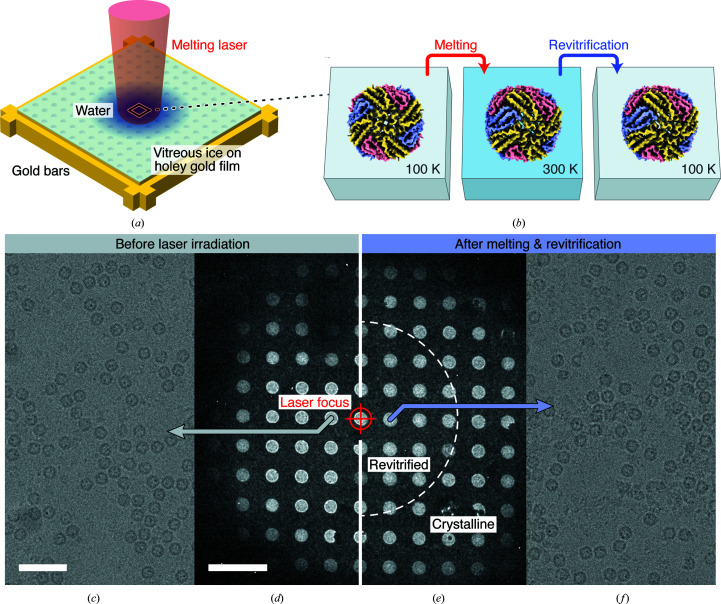
Rapid melting and revitrification of cryo samples: concept and experimental demonstration. (*a*) Illustration of the geometry of the cryo sample, which is prepared on a holey gold film supported by a gold mesh. The sample is irradiated *in situ* with a laser beam that is centered onto a grid square. (*b*) In the vicinity of the laser focus, the sample rapidly melts, allowing embedded particles to undergo equilibrium dynamics in the liquid phase. When the laser is switched off the sample rapidly revitrifies, trapping the particles, so that they can subsequently be imaged. (*c*)–(*f*) Micrographs of a cryo sample of apoferritin (*c*, *d*) and of the same sample after melting and revitrification with a 20 µs laser pulse (*e*, *f*). The laser focus is aligned to the central hole, which is marked with a crosshair. The outline of the revitrified area is indicated in (*e*) with a dashed semicircle. Adjacent regions have crystallized. The scale bars are 50 nm in (*c*) and 5 µm in (*d*).

**Figure 2:**
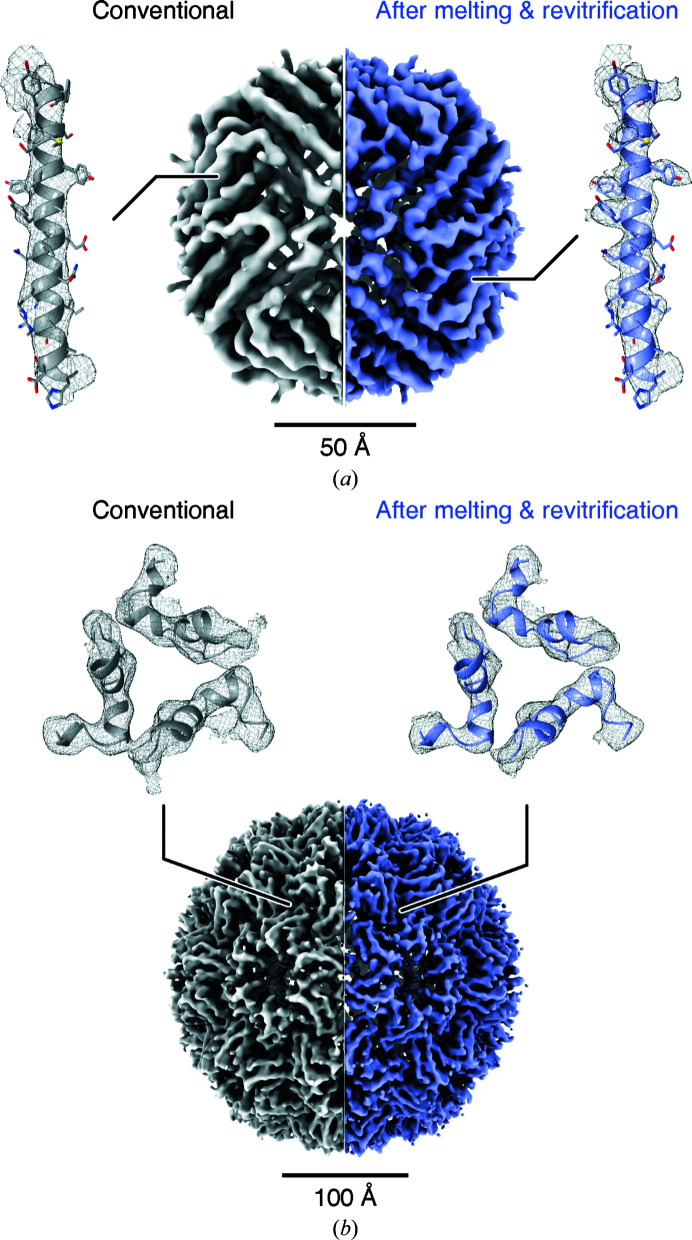
Comparison of single-particle reconstructions obtained from conventional and revitrified cryo samples. (*a*) Reconstructions of apoferritin from a conventional cryo sample (left, 4.57 Å resolution) and a melted and revitrified cryo sample (right, 4.25 Å resolution). Details are shown for the densities of an α-helix that has been fitted with PDB entry 6v21 (Wu *et al.*, 2020 ▸). (*b*) Single-particle reconstructions of CCMV from a conventional cryo sample (left, 4.98 Å resolution) and a melted and revitrified cryo sample (right, 5.20 Å resolution). Details of the densities of the capsid are shown in the vicinity of the quasi-threefold symmetry axis. The densities have been fitted with PDB entry 1cwp (Speir *et al.*, 1995 ▸). The structures obtained from conventional and revitrified samples are indistinguishable within the resolution of our instrument.

Cryo samples were prepared on UltrAuFoil R1.2/1.3 300 mesh grids (Quantifoil). The grids were rendered hydrophilic through 10 min of plasma cleaning in an ELMO glow-discharge system operating with negative polarity, 0.8 mA plasma current and 0.2 mbar residual air pressure. Approximately 3–3.5 µl of sample solution was applied to the foil side of the grids: either 1.70 mg ml^−1^ mouse heavy-chain apo­ferritin in 20 m*M* HEPES buffer with 300 m*M* sodium chloride at pH 7.5 (Fig. 1 ▸), 0.33 mg ml^−1^ mouse heavy-chain apoferritin in 20 m*M* Tris buffer with 150 m*M* sodium chloride at pH 7.4 (Figs. 2 ▸ and 3 ▸) or 11.5 mg ml^−1^ Cowpea chlorotic mottle virus (CCMV) in 0.1 *M* sodium acetate buffer with 1 m*M* Na_2_EDTA and 1 m*M* sodium azide at pH 5.0 (Figs. 2 ▸ and 3 ▸). The samples were plunge-frozen with a Vitrobot Mark IV (Thermo Fisher Scientific) that was operated at 100% relative humidity and a temperature of 22°C using a blotting time of 3–4 s. For imaging, the apoferritin and CCMV grids were loaded into either a Gatan 626 or a Gatan Elsa single-tilt cryo-transfer specimen holder, respectively.

Experiments were performed with a Jeol 2200FS transmission electron microscope that we have modified for time-resolved experiments as described previously (Olshin *et al.*, 2020 ▸). The microscope is operated at an accelerating voltage of 200 kV and is equipped with an in-column Omega-type energy filter and a K3 direct electron detector (Gatan). Microsecond laser pulses for *in situ* melting and revitrification of the sample are obtained by chopping the output of a continuous laser (532 nm) with an acousto-optic modulator. The laser is reflected from a mirror located above the upper pole piece of the objective lens and strikes the sample at close-to-normal incidence. The laser beam is focused to a spot size of 24 ± 1 µm FWHM as measured by a knife-edge scan in the sample plane.

Micrographs were zero-loss filtered with a 10 eV slit width. The electron detector was operated in counting mode to acquire 30-frame movies with a total dose of 60 electrons Å^−2^ and 2 and 3 s exposure times for apoferritin and CCMV, respectively. Micrographs were recorded with pixel sizes of 0.9750 and 0.8514 Å for apoferritin and CCMV, respectively. Defocus values were in the range 0.5–2.5 µm.

All images were corrected for the magnification distortion (Grant & Grigorieff, 2015 ▸), which we determined from micrographs of a gold calibration standard (Ted Pella, product No. 673) with the *mag_distortion_estimate*_1.0.1 script (Grant & Grigorieff, 2015 ▸). Single-particle reconstructions (see Supplementary Fig. S1 for workflows) were carried out with *cryoSPARC* version 3.2.0 (Punjani *et al.*, 2017 ▸). The conventional apoferritin data set consisted of 167 images. After patch motion correction and patch CTF estimation, 109 micrographs with CTF fits below 6.5 Å were selected. Following blob picking and inspection, particles were sorted into 30 classes, and five were used as templates to pick 146 763 particles from the micrographs. Of the picked particles, 81 332 were selected and sorted into 50 classes with a 160 Å circular mask. After removal of junk, 76 144 particles from 38 classes were selected and subjected to another round of 2D classification (50 classes, 140 Å mask). From this round of sorting, 55 183 particles across a total of 13 classes were selected. After *ab initio* reconstruction (*O* symmetry) and heterogeneous refinement (*O* symmetry) into two classes, the high-resolution class (49 885 particles) was homogeneously refined using *O* symmetry, resulting in a 4.57 Å resolution map.

The revitrified apoferritin data set was comprised of 100 micrographs taken from six melted and revitrified areas on one grid. After patch motion correction and patch CTF estimation, 70 images with CTF fits under 5.5 Å were chosen. Following blob picking and inspection, particles were sorted into 30 classes, and 27 were used as templates to pick 93 800 particles from the micrographs. Next, 58 792 particles were selected and sorted into 50 classes with a 160 Å circular mask. After removal of junk, 53 783 particles from 33 classes were chosen and subjected to another round of 2D classification (50 classes, 140 Å mask). A total of 40 classes, corresponding to 49 476 particles, were selected. After *ab initio* reconstruction (*O* symmetry) and heterogeneous refinement (*O* symmetry) into two classes, the high-resolution class (45 953 particles) was homogenously refined using *O* symmetry, resulting in a 4.25 Å resolution map.

The conventional CCMV data set consisted of 269 images. After patch motion correction and patch CTF estimation, 97 micrographs with CTF fits below 6.0 Å were selected. Following blob picking and inspection, particles were sorted into 30 classes, and 27 were used as templates to pick 36 783 particles from the micrographs. Of the picked particles, 6616 were selected and sorted into 50 classes with a 320 Å circular mask. After removal of junk, 6554 particles from 21 classes were selected. After *ab initio* reconstruction (*I* symmetry) and heterogeneous refinement (*I* symmetry) into two classes, the high-resolution class (3560 particles) was homogeneously refined using *I* symmetry, resulting in a 4.98 Å resolution map.

The revitrified CCMV data set consisted of 391 images taken from 22 melted and revitrified areas on seven grids. After patch motion correction and patch CTF estimation, 120 images with CTF fits below 6.5 Å were chosen. After blob picking and inspection, particles were sorted into 30 classes, and ten were used as templates to pick 42 201 particles from the micrographs. Of the picked particles, 7866 particles were selected and sorted into 50 classes with a 320 Å circular mask. After removal of junk, 7240 particles from 16 classes were selected. After *ab initio* reconstruction (*C*1 symmetry) and heterogeneous refinement (*I* symmetry) into two classes, the high-resolution class (5029 particles) was homogeneously refined using *I* symmetry, resulting in a 5.20 Å resolution map.

Single-particle reconstructions were visualized with *ChimeraX* (Goddard *et al.*, 2018 ▸). The volumes of apoferritin in Fig. 2(*a*) ▸ are displayed with contour levels of 0.20 and 0.15 for the conventional and revitrified samples, respectively. The molecular model from PDB entry 6v21 (Wu *et al.*, 2020 ▸) was placed into the density through rigid-body fitting. The details show the density within 3.3 Å of residues 13–42 of chain *C*. The volumes for CCMV in Fig. 2(*b*) ▸ are displayed with contour levels of 0.100 and 0.115 for the conventional and revitrified samples, respectively. The molecular model from PDB entry 1cwp (Speir *et al.*, 1995 ▸) was placed into the density through rigid-body fitting. Details are shown for the density within 3.3 Å of residues 76–86 and 142–153 of chains *A*, *B* and *C*.

**Figure 3:**
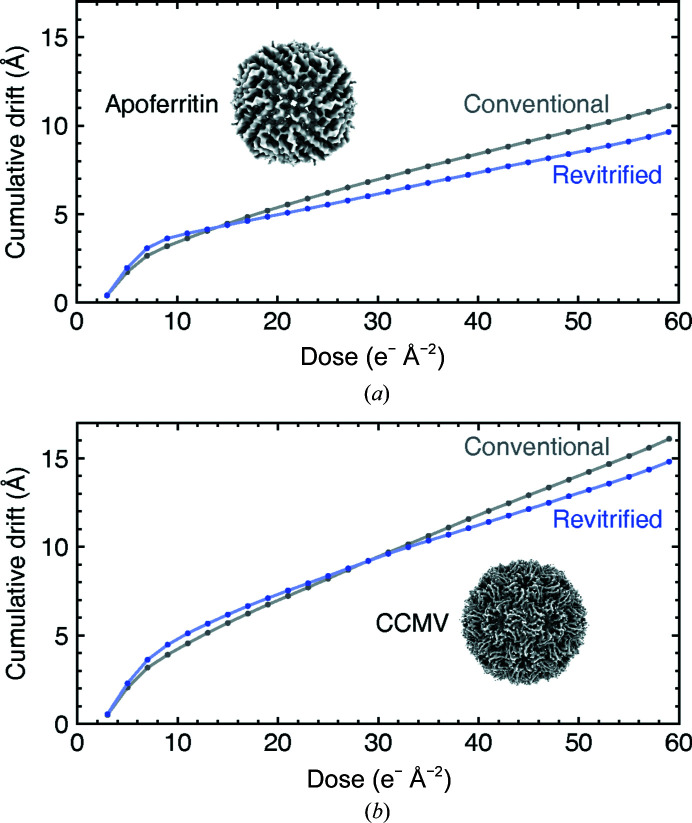
Comparison of sample drift in conventional and revitrified cryo samples. (*a*) Average cumulative specimen drift of apoferritin in a conventional cryo sample (gray) and a melted and revitrified cryo sample (purple). (*b*) Average cumulative specimen drift of CCMV in a conventional cryo sample (gray) and a melted and revitrified cryo sample (purple).

Drift trajectories of apoferritin and CCMV were determined with local motion correction in *cryoSPARC*. The average displacement of the particles as a function of dose (Fig. 3 ▸) was calculated using only the particles that were included in the reconstructions in Fig. 2 ▸. We note that if we include all particles the result is qualitatively the same.

## 3. Results and discussion

Figs. 1 ▸(*a*) and 1 ▸(*b*) illustrate the sample geometry and experimental approach. Cryo samples are prepared on UltrAuFoil R1.2/1.3 300 mesh gold grids (Russo & Passmore, 2014 ▸) and the melting laser (532 nm, 24 ± 1 µm spot size in the sample plane) is centered on a grid square (Fig. 1 ▸ *a*). Under illumination with a 20 µs laser pulse, the sample rapidly melts in the vicinity of the laser focus and subsequently revitrifies, arresting the motions of the embedded particles (Fig. 1 ▸ *b*).

Figs. 1 ▸(*c*)–1 ▸(*f*) display micrographs of a typical experiment with a cryo sample of mouse apoferritin. Fig. 1 ▸(*d*) shows a low-magnification view of the grid square before laser irradiation and Fig. 1 ▸(*c*) shows a micrograph collected from the hole marked with a gray arrow. The sample is then illuminated *in situ* with a 20 µs laser pulse (205 mW), with the laser beam aligned to the central hole, which is marked with a crosshair. As indicated in Fig. 1 ▸(*e*), a circular area around the center of the laser focus has melted and revitrified (dashed semicircle). In contrast, the adjacent regions, in which the temperature has remained below the melting point of water, have crystallized (Voss *et al.*, 2021*a* ▸). We then collect micrographs from holes within the revitrified area, such as that in Fig. 1 ▸(*f*), which reveals intact apoferritin particles. We note that in order to ensure a comparable temperature evolution in all melting and revitrification experiments, the laser power (typically about 165 mW) was always adjusted to obtain a revitrified region of similar size to that in Fig. 1 ▸(*e*) (Voss *et al.*, 2021*a* ▸). We note that while aligning the laser the sample is exposed to a dose of about 10^−3^ electrons Å^−2^ before it is melted and revitrified. This dose is too low to induce any detectable amount of beam damage.

Single-particle reconstructions confirm that the melting and revitrification process preserves the structure of apoferritin (Wu *et al.*, 2020 ▸). Fig. 2 ▸(*a*) compares the density map obtained from a conventional cryo sample (left, 4.57 Å resolution) with that obtained after melting and revitrification (right, 4.25 Å resolution). We note that the resolution is consistent with that previously obtained on a similar instrument (Kayama *et al.*, 2021 ▸). Individual helices are clearly resolved in both reconstructions. As evident in the details shown on the left and right, some side-chain density is visible, which is slightly more pronounced in the higher-resolution map obtained from the revitrified samples. Within the resolution afforded by our instrument, the structures are indistinguishable. This result is confirmed by a second set of experiments on cryo samples of CCMV, an icosahedral plant virus (Speir *et al.*, 1995 ▸; Vriezema *et al.*, 2005 ▸; Fig. 2 ▸ *b*). We obtain near-identical reconstructions of CCMV with resolutions of 4.98 and 5.20 Å for the conventional (left) and revitrified (right) cryo samples, respectively. Secondary structural elements of the viral capsid are well resolved, which is also evident in the details of the density in the vicinity of the quasi-threefold symmetry axis. We note that the viral RNA inside the capsid is disordered and therefore is not resolved. We conclude that the melting and revitrification process leaves the particles intact and that within the spatial resolution of our experiment it does not alter their structure.

Evidently, rapid melting and revitrification does not expose the particles to any mechanical forces or other processes that damage their structure. This conclusion is supported by a consideration of the different elements of the experiment, including the nature of the interaction between the laser beam and the particles, the melting and the revitrification steps, as well as the interactions that the particles encounter while the sample is liquid.

The interaction of the particles with the 532 nm laser beam is only indirect, since neither the particles nor the vitreous ice film absorb visible light, which prevents any type of photodamage. Instead, the laser heats the holey gold film of the specimen grid, which then melts the vitreous ice through rapid heat diffusion. We have previously shown that the sample temperature can be controlled to prevent heat denaturation of the particles. In particular, evaporative cooling provides negative feedback that stabilizes the sample temperature (Voss *et al.*, 2021*a* ▸,*b* ▸).

While the sample is liquid, the particles may interact with the interface between the water film and the vacuum of the microscope. Interactions with the air–water interface have previously been shown to lead to particle denaturation (D’Imprima *et al.*, 2019 ▸). However, the number of such collisions on the timescale of our experiment, tens of microseconds, is significantly lower than during the plunge-freezing process, where the time between blotting and vitrification is of the order of a second. Furthermore, it has previously been concluded that during this time a layer of unraveled proteins forms at the aqueous surface that protects particles from reaching the interface (Yoshimura *et al.*, 1994 ▸; Glaeser, 2018 ▸). We therefore expect the number of collisions that lead to denaturation to be small as long as the thickness of the sample remains large enough.

The rapid revitrification of the sample after the laser pulse should certainly not damage the particles, since it is well established that vitrification preserves the structure of a protein. In fact, the significantly higher cooling rate in our experiment (Voss *et al.*, 2021*b* ▸), compared with that typically reached during plunge-freezing (Frank, 2017 ▸; Shaikh *et al.*, 2009 ▸), should be better suited to trap the room-temperature structure of proteins. The melting step resembles the re­vitrification process in that it occurs on the same timescale of a few microseconds, just with the temperature evolution reversed (Voss *et al.*, 2021*b* ▸). One notable difference, however, is that cubic ice forms during the early stages of laser heating, which only melts once the temperature exceeds 273 K (Voss *et al.*, 2021*a* ▸). While the formation of cubic ice crystals could conceivably damage the proteins, this does not appear to be the case. In fact, it has recently been shown that the structure of proteins in devitrified cryo samples is unchanged and that devitrification can even be used to reduce sample drift and improve resolution (Wieferig *et al.*, 2021 ▸).

While we conclude that the structure of the proteins is not altered, an important related question is whether melting and revitrification changes the properties of the ice. In particular, it is important to establish how it affects the beam-induced motion that occurs during imaging and that limits the obtainable spatial resolution. Such motion arises when mechanical stresses in the vitreous ice, which have built up during plunge-freezing, are released under electron irradiation (Russo & Passmore, 2014 ▸; Naydenova *et al.*, 2020 ▸; Wright *et al.*, 2006 ▸; Brilot *et al.*, 2012 ▸; Zheng *et al.*, 2017 ▸; Engstrom *et al.*, 2021 ▸; Thorne, 2020 ▸). Fig. 3 ▸(*a*) shows that the average cumulative drift of the apoferritin particles, which is displayed as a function of the electron dose, follows a typical behavior for both the conventional (gray) and revitrified (purple) cryo samples. They initially drift quickly, before slowing to a lower and constant drift rate after a dose of 10–20 electrons Å^−2^. The revitrified samples exhibit a slightly larger initial drift but a smaller asymptotic drift rate. Their average cumulative drift therefore drops below that of the conventional samples after 15 electrons Å^−2^. It is 1.5 Å lower at a dose of 60 electrons Å^−2^, with a total drift of 10 Å. Qualitatively similar, although less pronounced, differences are observed for the beam-induced motion of the CCMV samples (Fig. 3 ▸ *b*). The cumulative drift of the revitrified samples drops below that of the conventional samples after 30 electrons Å^−2^ and is 1.3 Å lower at a dose of 60 electrons Å^−2^, with a total drift of 15 Å.

We conclude that revitrified samples exhibit comparable amounts of beam-induced motion to conventional samples, an observation that can shed new light on the mechanism that causes a buildup of stress in the ice during vitrification. It has been proposed that this stress results from the large temperature difference between the holey gold film and the grid bars during plunge-freezing. Because of their large heat capacity, the bars cool more slowly and thus exert a compressive force on the vitreous ice film once they contract (Engstrom *et al.*, 2021 ▸; Thorne, 2020 ▸). Since melting and revitrification is highly localized and the grid bars remain at cryogenic temperatures throughout the entire process, this mechanism alone cannot easily explain the beam-induced motion of the revitrified samples (Voss *et al.*, 2021*a* ▸,*b* ▸). Our results are also seemingly at odds with the previous observation that drift increases with vitrification speed, which has been ascribed to the fact that faster cooling provides less time for stresses in the ice to dissipate during plunge-freezing (Wu *et al.*, 2021 ▸; Engstrom *et al.*, 2021 ▸). Since the cooling rate during revitrification is almost two orders of magnitude higher, one would therefore expect the beam-induced motion to become even more pronounced. We speculate that this is not the case since revitrification removes the stresses that arise from large-scale deformations of the entire grid during plunge-freezing (Engstrom *et al.*, 2021 ▸; Thorne, 2020 ▸), which compensates for the additional local stress induced by the high cooling rate.

## 4. Conclusion

Our experiments demonstrate that the rapid melting and revitrification of cryo samples leaves the embedded particles intact, providing a central piece of evidence that our approach is suitable to study the dynamics of proteins on the microsecond timescale. We find that proteins do not undergo structural changes within the spatial resolution afforded by our instrument. However, subtle structural differences, such as a side chain adopting a different conformation, may become apparent at higher resolution, a possibility that we have begun to explore. Temperature-resolved cryo-EM experiments have long established that for some proteins different conformational ensembles are obtained when the sample is cooled to a lower temperature before vitrification (Fischer *et al.*, 2010 ▸; Chen *et al.*, 2019 ▸). Functional states present at physiological temperatures may even be depopulated during the plunge-freezing process if the conformational transitions involved are fast relative to the cooling rate (Mehra *et al.*, 2020 ▸). Melting and revitrification may provide a tool to investigate such effects systematically and even repopulate high-temperature states that are inaccessible at lower vitrification speeds. We also expect that the significantly faster cooling rate in our experiments should lead to a higher glass transition temperature (Angell, 2008 ▸) and thus cause subtle changes in the structure of the water network surrounding the proteins, which should become evident at atomic resolution (Nakane *et al.*, 2020 ▸; Yip *et al.*, 2020 ▸).

Our experiments also open up new possibilities for studying the vitrification process in real time (Olshin *et al.*, 2021 ▸) and how it leads to the buildup of stress that results in beam-induced motion. By suitably modulating the laser power, the cooling rate can be varied by more than three orders of magnitude, and the temperature evolution of the sample can be precisely controlled. This has been difficult to achieve with plunge-freezing, where the complex fluid dynamics of the cryogen and its vapor phase as well as the heterogeneous heat-transfer properties of the sample lead to widely varying vitrification speeds, even across a single specimen grid (Wu *et al.*, 2021 ▸). Even though we observe only small differences in the beam-induced motion of revitrified samples, our initial results suggest that it may be possible to release stress through irradiation with a sequence of laser pulses.

In the future, we envision using the microsecond melting and revitrification approach to study the dynamics of proteins, as outlined previously (Voss *et al.*, 2021*b* ▸). Several approaches are available for initiating dynamics. The intensity of the melting laser can be increased to heat the sample to elevated temperatures and thus induce a temperature jump, or a second laser pulse can be employed to directly trigger a photoactivated process. Alternatively, caged compounds can be used to provide a range of biomimetic stimuli by inducing a pH jump (Gutman, 1984 ▸) or releasing ATP, ions or even small peptides (Ellis-Davies, 2007 ▸; Shigeri *et al.*, 2001 ▸). Con­veniently, these compounds can be released with the sample still in the frozen state. Since the matrix of vitreous ice prevents motion of the proteins, dynamics will only occur once the sample is melted. By melting different areas of the sample for different amounts of time, reconstructions can then be obtained of several time points, so as to capture the complete structural evolution of the particles.

## 5. Data availability

The cryo-EM maps presented in this manuscript have been deposited in EMDB with the following accession codes: conventional apoferritin, EMD-14767; revitrified apoferritin, EMD-14768; conventional CCMV, EMD-14769; revitrified CCMV, EMD-14772. The corresponding micrographs have been deposited in EMPIAR with the following accession codes: conventional apoferritin, EMPIAR-11016; revitrified apoferritin, EMPIAR-11017; conventional CCMV, EMPIAR-11018; revitrified CCMV, EMPIAR-11019. All other data that support the findings of this study are available from the corresponding author upon reasonable request.

## Supplementary Material

EMDB reference: apoferritin, conventional, EMD-14767

EMDB reference: revitrified, EMD-14768

EMDB reference: CCMV, conventional, EMD-14769

EMDB reference: revitrified, EMD-14772

Supplementary Figure S1. DOI: 10.1107/S205979832200554X/vo5009sup1.pdf
