# Supplementary material for: Microsecond melting and revitrification of cryo samples: protein structure and beam-induced motion
Source: Acta Crystallogr D Struct Biol. 2022 Jun 14;78(Pt 7):883–9. doi: 10.1107/S205979832200554X (PMC9248841; doi:10.1107/S205979832200554X)
Supplement: Supplementary file 1 [file d-78-00883-sup1.pdf]

STRUCTURAL BIOLOGY

Volume 78 (2022)

Supporting information for article:

Microsecond melting and revitrification of cryo samples: protein structure and beam-Induced motion

Oliver F. Harder, Jonathan M. Voss, Pavel K. Olshin, Marcel Drabbels and Ulrich J. Lorenz

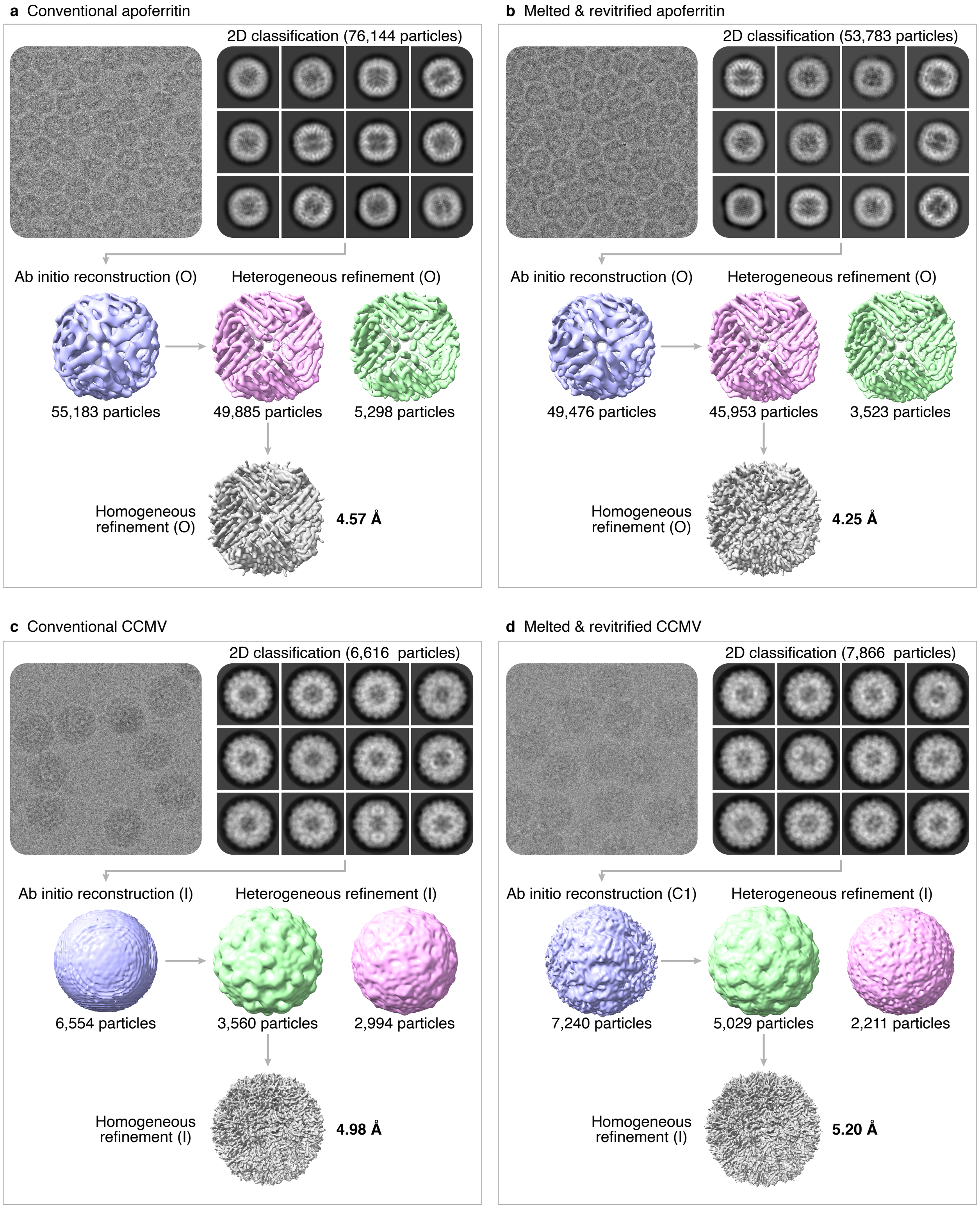

Figure S1. Workflows for single-particle reconstructions of apoferritin (**a**,**b**) and CCMV (**c**,**d**) from conventional and revitrified samples.
